# Janus VSSi as an efficient 2D electrode for Sb_2_C_3_ with tunable Schottky contact: a first-principles study

**DOI:** 10.1039/d6ra03814j

**Published:** 2026-07-06

**Authors:** Ho Kim Dan, Huynh Thi Phuong Thuy, Le Phuong Long, Le Phuoc Dinh, Nguyen D. Hien, Khang D. Pham

**Affiliations:** a Optical Materials Research Group, Science and Technology Advanced Institute, Van Lang University Ho Chi Minh City Vietnam hokimdan@vlu.edu.vn; b Faculty of Applied Technology, Van Lang School of Technology, Van Lang University Ho Chi Minh City Vietnam; c Thu Dau Mot University Ho Chi Minh City Vietnam; d Center of Scientific Research and Application, Lac Hong University No. 10 Huynh Van Nghe Str Tran Bien Ward Dong Nai Province Vietnam phuonglong@lhu.edu.vn; e Faculty of Electricity, Electronics and Material Technology, University of Sciences, Hue University Hue Vietnam; f Nha Trang Center Ethnic Minority Pre-university School No. 46 Nguyen Thien Thuat Str. Nha Trang Ward Khanh Hoa Province Vietnam; g Institute of Research and Development, Duy Tan University Da Nang 550000 Vietnam phamdinhkhang@duytan.edu.vn; h School of Engineering & Technology, Duy Tan University Da Nang 550000 Vietnam

## Abstract

Two-dimensional (2D) metal/semiconductor van der Waals (vdW) heterostructures have attracted significant attention for next-generation nanoelectronic devices due to their tunable interfacial properties. In this study, first-principles calculations are employed to comprehensively explore the structural stability, electronic behavior, and contact properties of the Janus VSSi/Sb_2_C_3_ heterostructure. Our results demonstrate that the heterostructure is energetically, mechanically, and dynamically stable. Depending on the interfacial configuration, distinct contact behaviors are observed, where the Si-terminated interface forms an ohmic contact, while the S-terminated interface exhibits an n-type Schottky contact with a relatively low barrier height, facilitating efficient electron injection. The absence of significant metal-induced gap states indicates weak Fermi level pinning, allowing the Schottky barrier to closely follow the Schottky–Mott limit. Furthermore, the tunneling probability and tunneling-specific resistivity confirm the presence of low contact resistance and efficient carrier transmission. Notably, the application of an external electric field enables effective modulation of the Schottky barrier and induces a transition between n-type and p-type contacts *via* band-edge engineering. These results establish Janus VSSi as a promising two-dimensional metallic electrode and highlight the VSSi/Sb_2_C_3_ heterostructure as a potential building block for future nanoelectronic and optoelectronic devices.

## Abstract 2

Two-dimensional (2D) metal/semiconductor van der Waals (vdW) heterostructures have attracted significant attention for next-generation nanoelectronic devices due to their tunable interfacial properties.

## Graphical abstract

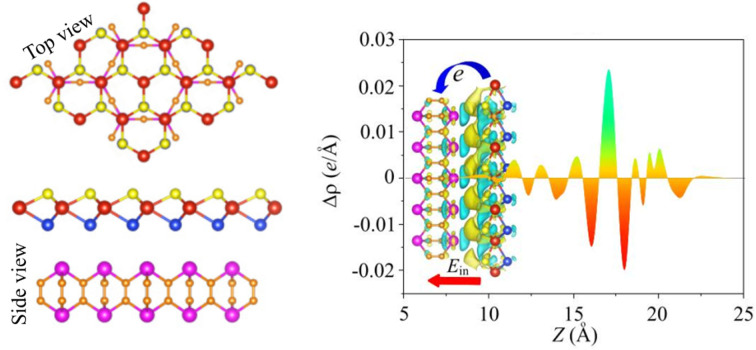

## 1 Introduction

Recent advances in two-dimensional (2D) materials have opened new opportunities for designing high-performance nanoelectronic and optoelectronic devices. To date, various types of 2D materials have been proposed computationally and synthesized experimentally, including graphene,^1^ dichalcogenides (TMDs),^2^ h-BN,^3^ phosphorus,^4^ as well as emerging classes like MXenes^5^ and Janus monolayers.^6,7^ Owing to their reduced dimensionality and unique electronic structures, these materials exhibit a broad range of properties from metallic and semimetallic to semiconducting and insulating, making them highly versatile for next-generation device architectures.^8,9^

Beyond individual monolayers, the vertical stacking of distinct 2D materials to form van der Waals (vdW) heterostructures offers a powerful platform for tailoring novel physical properties and device functionalities.^10,11^ In contrast to conventional heterostructures, vdW systems are largely free from lattice-matching constraints and dangling bond effects, enabling the integration of dissimilar materials with minimal interfacial defects and structural distortion.^12^ Among various configurations, metal/semiconductor vdW interfaces are of particular importance, as they play a decisive role in determining device performance, especially in terms of carrier injection efficiency and contact resistance.^13,14^ In conventional metal–semiconductor contacts, the formation of Schottky barriers often limits carrier transport, leading to increased contact resistance.^15,16^ In vdW heterostructures, by contrast, the weak interlayer interaction can effectively suppress the formation of such interface states, offering a promising route to engineer tunable and potentially low-resistance contacts.^17,18^ Several metal/semiconductor interfaces based on 2D materials have been extensively investigated through both experimental measurements and first-principles calculations. For instance, contacts between graphene and TMDs^19,20^ have been shown to exhibit tunable Schottky barriers depending on the interface configuration and external modulation, while conventional 3D metals in contact with TMDs have been widely studied to achieve either Schottky or near-Ohmic behavior.^21,22^ More recently, a variety of emerging two-dimensional metallic materials have been investigated and demonstrated promising performance as electrodes when integrated with other 2D semiconductors, including Goldene,^23^ MoSH^24,25^ and NbS(Se)_2_ TMDs.^26,27^

Very recently, Janus VSSi monolayers have been predicted computationally as promising contact electrode materials.^28^ Owing to their intrinsic structural asymmetry and excellent stability, Janus VSSi can be integrated with other 2D semiconductors like InSe to form vdW heterostructures with high tunneling probability and low tunneling-specific resistivity.^29^ In parallel with the development of 2D metallic materials, considerable efforts have also been devoted to exploring novel two-dimensional semiconductors with suitable band gaps and high carrier mobility. Among these, the Sb_2_C_3_ monolayer, belonging to the hexagonal M_2_C_3_ (M = As, Sb, Bi) family, has been theoretically predicted as a promising semiconductor with desirable electronic properties.^30^ In addition, the Sb_2_C_3_ monolayer has been identified as a potential candidate for gas sensing applications, particularly for the detection of environmentally toxic gases.^31^

Despite these promising properties, the interfacial characteristics and contact behavior of Sb_2_C_3_ when integrated with Janus VSSi metallic electrodes remain largely unexplored. In particular, a detailed understanding of band alignment, charge transfer, and Schottky barrier formation at the Janus VSSi/Sb_2_C_3_ interface is still lacking. Therefore, a comprehensive first-principles investigation is highly desirable to elucidate the contact nature and evaluate the feasibility of achieving low-resistance contacts and efficient carrier injection in such vdW heterostructures.

## 2 Computational details

Quantum ESPRESSO^32,33^ was employed to carry out density functional theory calculations. The exchange-correlation potential was modeled using the PBE functional within the GGA framework.^34,35^ Core-valence interactions were described through the PAW formalism.^36^ To obtain converged results, the plane-wave expansion was adopted at a kinetic-energy cutoff of 520 eV. A *Γ*-centered Monkhorst–Pack *k*-point mesh of 12 × 12 × 1 was employed for Brillouin-zone integrations. The convergence of this *k*-point sampling was carefully examined, and the results are presented in Fig. S1 of the SI. The valence electronic configurations explicitly considered in the calculations are V (3d^3^4s^2^), S (3s^2^3p^4^), Si (3s^2^3p^2^), Sb (5s^2^5p^3^), and C (2s^2^2p^2^). To obtain more reliable band-gap values for the investigated 2D monolayers, the screened hybrid functional proposed by Heyd–Scuseria–Ernzerhof (HSE)? was employed. To mitigate periodic image interactions and artificial electrostatics, we implemented a 30 Å vacuum layer and a dipole correction, respectively. Furthermore, long-range dispersion was captured *via* the DFT-D3 method.^37^ The system reached its ground state once the Hellmann–Feynman forces dropped below 0.01 eV Å^−1^ and energy fluctuations stayed within 10^−8^ eV. The stability of the investigated structures was assessed from both dynamical and thermal perspectives. Dynamical stability was verified through DFPT-based phonon calculations? employing a 3 × 3 × 1 supercell. Thermal stability was subsequently examined using AIMD simulations within the canonical ensemble (NVT), where a Nosé–Hoover thermostat maintained the system temperature at 300 K. The simulation time and integration step were set to 8 ps and 1 fs, respectively.

## 3 Results and discussion

**Fig. 1:**
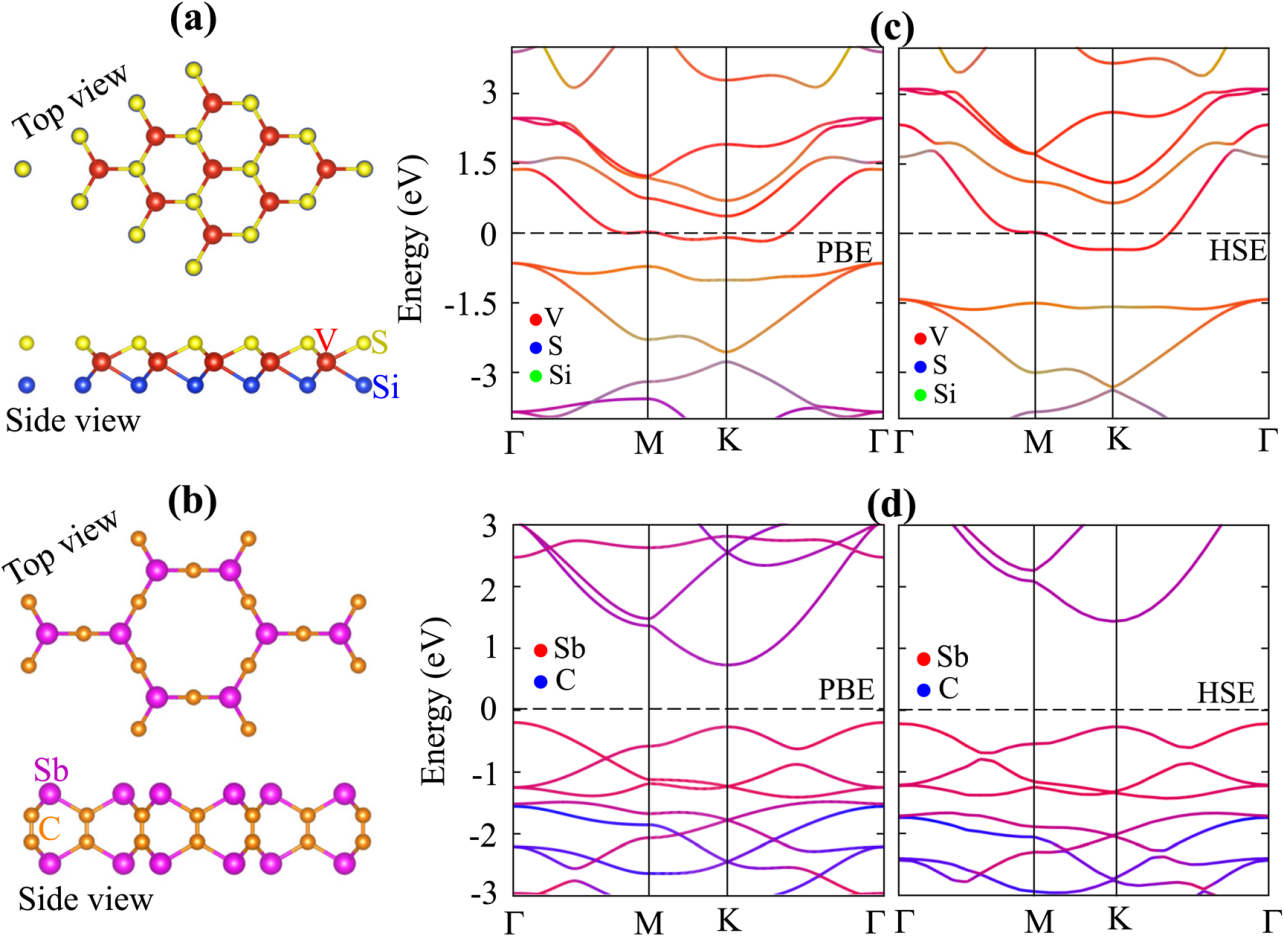
Atomic structures of (a) VSSi, (b) Sb_2_C_3_ monolayers. Band structures using PBE and HSE functionals of (c) VSSi and (d) Sb_2_C_3_ monolayers. The Fermi level is aligned at 0 eV and indicated by a black dashed line.

To gain fundamental insights into the properties of the constituent materials, we first examine the structural, electronic, and dynamical characteristics of monolayers, as shown in Fig. 1. The optimized atomic structures of Janus VSSi and Sb_2_C_3_ monolayers are presented in Fig. 1(a) and (b), respectively. The VSSi monolayer exhibits a typical Janus configuration with broken out-of-plane symmetry, while Sb_2_C_3_ adopts a hexagonal lattice structure. In the VSSi monolayer, each V atom is covalently bonded to S atoms on one side and Si atoms on the opposite side. Similarly, in the Sb_2_C_3_ monolayer, each C atom is coordinated with neighboring Sb atoms, forming a hexagonal framework composed of six Sb and six C atoms, as illustrated in Fig. 1(b). This arrangement results in a stable two-dimensional network with strong in-plane bonding. The optimized geometries reveal lattice parameters of 3.58 and 6.40 Å for the VSSi and Sb_2_C_3_ monolayers, respectively. These values are comparable to those previously reported, confirming the reliability of the present computational approach.^29,30^ Furthermore, the band structures calculated using both the PBE and HSE06 functionals, as shown in Fig. 1(c), consistently reveal the metallic nature of the VSSi monolayer, evidenced by the presence of a band crossing the Fermi level. In contrast, the Sb_2_C_3_ monolayer exhibits an indirect semiconducting character with a band gap of 0.93 eV at the PBE level, which increases to 1.65 eV when the HSE06 functional is employed, as shown in Fig. 1(d). The enlarged band gap obtained from the HSE06 calculation mainly originates from the upward shift of the conduction bands, while the overall band dispersion remains nearly unchanged. Notably, both PBE and HSE06 functionals consistently predict that the valence band maximum (VBM) and conduction band minimum (CBM) are located at the *Γ* and *K* points, respectively, confirming the indirect-band-gap nature of the Sb_2_C_3_ monolayer. Since both PBE and HSE06 functionals yield the same qualitative electronic characteristics, including the metallic nature of VSSi and the indirect-band-gap feature of Sb_2_C_3_, the PBE functional is adopted for the subsequent calculations owing to its significantly lower computational cost while maintaining reliable predictions of the electronic properties.

**Fig. 2:**
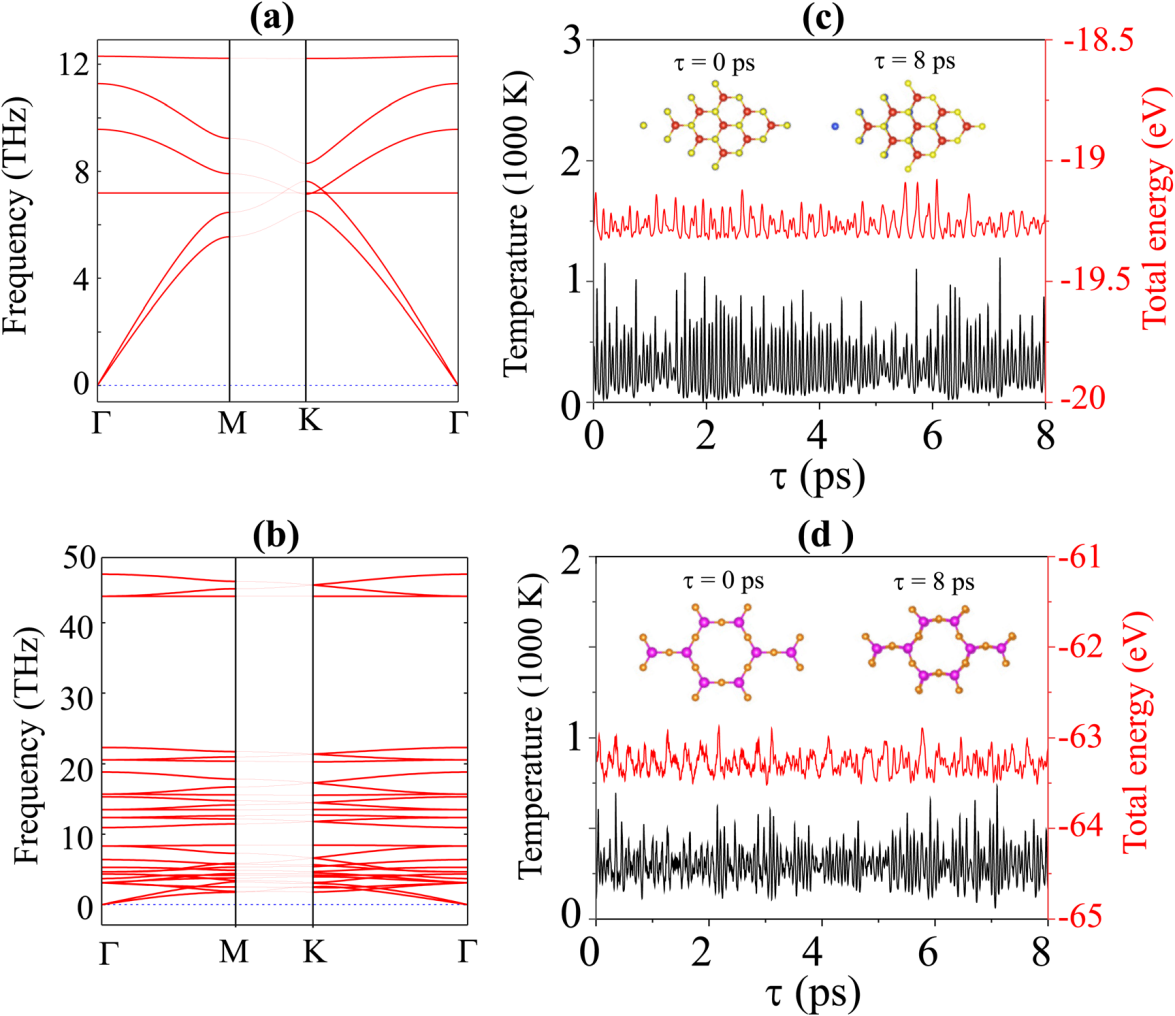
Atomic structures of (a) VSSi, (b) Sb_2_C_3_ monolayers. Band structures of (c) VSSi and (d) Sb_2_C_3_ monolayers. Phonon dispersion curves of (e) VSSi and (f) Sb_2_C_3_ monolayers.

To gain further insight into the stability of the investigated monolayers, phonon spectra were calculated, and the corresponding results are presented in Fig. 2(a,b). The absence of imaginary frequencies throughout the Brillouin zone confirms the dynamical stability of both VSSi and Sb_2_C_3_ monolayers, suggesting their feasibility for experimental realization. Furthermore, AIMD simulations were carried out at 300 K to assess their thermal stability under ambient conditions. As shown in Fig. 2(c and d), the total energies of both monolayers fluctuate only slightly around their equilibrium values during the simulation, without exhibiting any abrupt variation. Moreover, no noticeable structural reconstruction or bond breaking is observed in the final configurations. These results further verify the excellent thermal stability of the VSSi and Sb_2_C_3_ monolayers at room temperature.

**Fig. 3:**
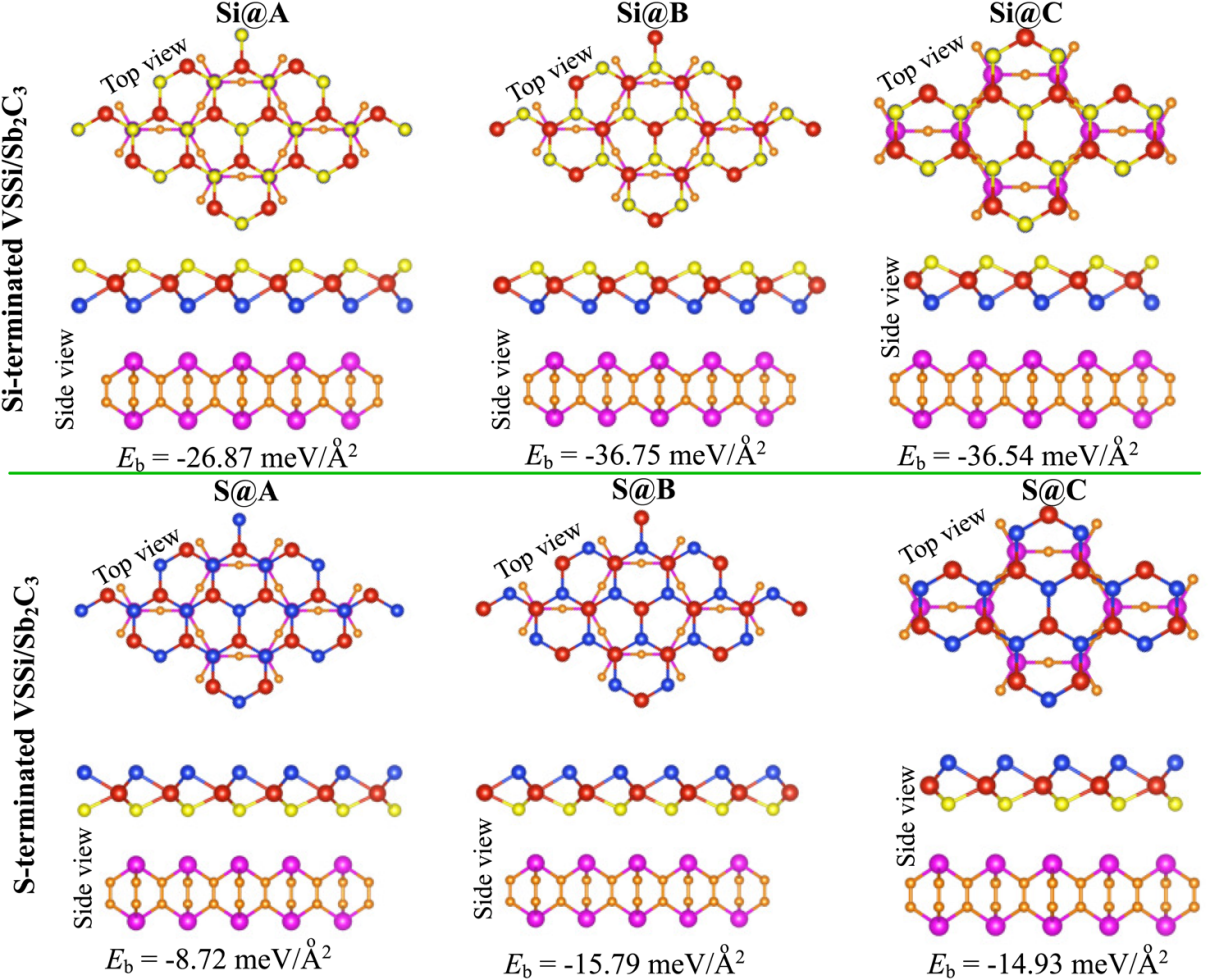
Geometric optimization of atomic structures of the VSSi/Sb_2_C_3_ with different stacking sides of Si-terminated and S-terminated surfaces.

To elucidate the interfacial characteristics of the heterostructure, the Janus VSSi/Sb_2_C_3_ heterostructure were constructed by vertically stacking the two constituent monolayers. Due to the intrinsic out-of-plane asymmetry of the Janus VSSi layer, two inequivalent interfacial configurations naturally emerge, depending on whether the Si-terminated or S-terminated surface is brought into contact with the Sb_2_C_3_ sheet, as illustrated in Fig. 3. To construct the VSSi/Sb_2_C_3_ vdW heterostructure, a supercell was generated by matching a 
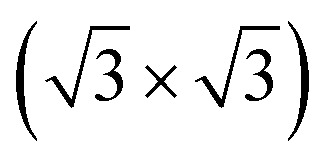
 VSSi supercell with a (1 × 1) Sb_2_C_3_ unit cell. Based on the optimized lattice constants of the isolated monolayers, the resulting heterostructure was built using an in-plane lattice parameter of 6.30 Å, corresponding to the average lattice constant of the two constituents. Consequently, the VSSi layer undergoes a slight tensile strain, whereas the Sb_2_C_3_ layer experiences a small compressive strain. The lattice mismatch is estimated to be only 1.60%, which is expected to have a negligible influence on the intrinsic electronic properties of the constituent layers. For each termination, three representative stacking patterns were considered, denoted as Si(S)@A, Si(S)@B, and Si(S)@C, corresponding to different relative atomic registries between the two layers. The optimized interlayer separations for the Si-terminated configurations are found to be 2.89, 2.77, and 2.78 Å for Si@A, Si@B, and Si@C, respectively. In contrast, the S-terminated counterparts exhibit larger equilibrium distances of 3.34, 3.27, and 3.30 Å for S@A, S@B, and S@C, respectively. This noticeable difference in interlayer spacing suggests a stronger interfacial interaction in the Si-terminated configurations compared to the S-terminated ones. In addition, these interlayer spacings fall within the typical range reported for vdW heterostructures, such as GeS/WS_2_,^38^ SiS/WSSe,^39^ MoSi_2_N_4_-based,^40^ TMDs-based,^41^ and MoSSe-based^42,43^ systems, further confirming the vdW nature of the interface.

Furthermore, we evaluate the energetic stability of the proposed configurations by calculating the binding energy, which provides quantitative insight into the strength of interlayer coupling and the feasibility of experimental realization. The binding energy of the heterostructure is computed using:1
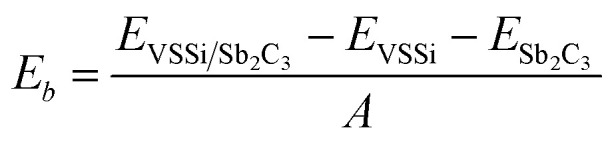
Here, *E*_VSSi/Sb_2_C_3__, *E*_VSSi_, and *E*_Sb_2_C_3__ denote the total energies of the heterostructure, the isolated VSSi monolayer, and the isolated Sb_2_C_3_ monolayer, respectively, while *A* represents the interfacial area of the supercell. The obtained negative binding energy demonstrates that the heterostructure formation process is energetically favorable and stable. All the binding energies of the Si-terminated and S-terminated VSSi/Sb_2_C_3_ heterostructure are illustrated in Fig. 3. The calculated binding energies reveal that, for the VSSi/Sb_2_C_3_ heterostructure with the Si-terminated surface, the Si@B stacking configuration is the most energetically stable, as evidenced by its lowest *E*_*b*_ value of −36.75 meV Å^−2^. For the S-terminated interface, the S@B stacking configuration is also identified as the most stable among the considered arrangements.

**Fig. 4:**
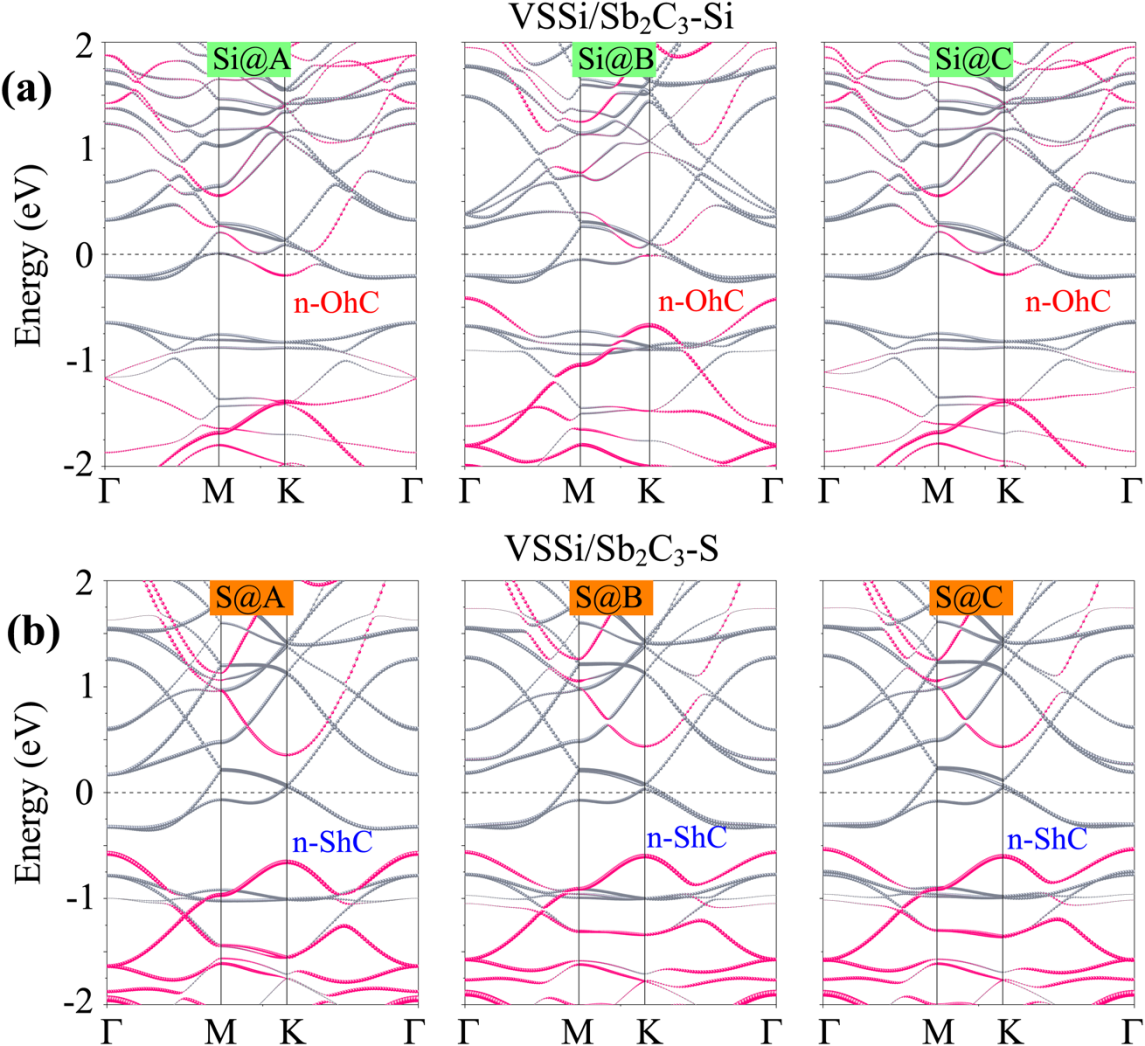
Orbital-projected band structures of the VSSi/Sb_2_C_3_ vdW heterostructure corresponding to various stacking configurations: (a) Si-terminated and (b) S-terminated interfaces. The purple and gray colors represent the orbital contributions from the Sb_2_C_3_ and VSSi layers, respectively.

Building on the optimized structures, we next examine the projected band structures to elucidate the interfacial electronic properties of the VSSi/Sb_2_C_3_ heterostructures for both the Si-terminated and S-terminated surfaces, as illustrated in Fig. 4. Notably, upon stacking metallic VSSi with semiconducting Sb_2_C_3_, the heterostructure preserves metallic characteristics, with states crossing the Fermi level predominantly originating from the VSSi layer. Depending on the relative alignment between the Fermi level of VSSi and the band edges of Sb_2_C_3_, different contact behaviors can be identified. Specifically, when the Fermi level lies within the band gap but closer to either the CBM or VBM, an n-type or p-type Schottky contact (ShC) is formed, respectively. In contrast, if the Fermi level intersects the CBM or VBM, the Schottky barrier vanishes, leading to the formation of an ohmic contact (OhC) with efficient carrier injection. As depicted in Fig. 4(a), the VSSi/Sb_2_C_3_ heterostructure with the Si-terminated surface forms an OhC contact because the Fermi level intersects the CBM of the Sb_2_C_3_ layer, resulting in a vanishing Schottky barrier and facilitating efficient electron injection. In contrast, the VSSi/Sb_2_C_3_ heterostructure with the S-terminated surface leads to the formation of a ShC contact owing to the misalignment between the Fermi level and the band edges of Sb_2_C_3_, where the Fermi level resides within the band gap, giving rise to a finite Schottky barrier. In the ShC contact, the Schottky barrier height (SBH) can be estimated based on the Schottky–Mott rule. For an n-type contact, the SBH is defined as:

2 *Φ*_n_ = *E*_CBM_ − *E*_F_

where the conduction-band edge of the semiconductor is represented by *E*_CBM_, whereas *E*_F_ corresponds to the Fermi energy of the heterostructure. Similarly, for a p-type contact, the SBH is given by:

3 *Φ*_p_ = *E*_F_ − *E*_VBM_

where *E*_VBM_ is the VBM of the semiconductor. The calculated *Φ*_n_ and *Φ*_p_ for the VSSi/Sb_2_C_3_ heterostructure with the S-terminated surface are 0.36 and 0.65 eV for the S@A configuration, 0.44 and 0.60 eV (S@B), and 0.43 and 0.60 eV (S@C), respectively. These results indicate that the n-type Schottky barrier is consistently lower than the p-type counterpart, suggesting that electron injection is more favorable than hole injection in the S-terminated interfaces. Accordingly, the VSSi/Sb_2_C_3_ heterostructure with the S-terminated surface forms an n-type Schottky contact, which is beneficial for device applications, as the reduced electron barrier enables efficient carrier injection.

More interestingly, the VSSi/Sb_2_C_3_ heterostructure exhibits competitive performance when compared with previously reported 2D metal/2D semiconductor vdW interfaces. Specifically, the calculated SBH falls within the typical range reported for graphene-based heterostructure,^44–46^ germanene/MoSSe,^47^ Goldene/MXene^23,48^ and MBene/MoSSe.^49^ This suggests that the VSSi/Sb_2_C_3_ interface can serve as an efficient contact platform with improved carrier injection efficiency. In addition, the formation of either Schottky or ohmic contact depending on the Si-terminated or S-terminated interface in the VSSi/Sb_2_C_3_ heterostructure highlights the high tunability of contact characteristics *via* interfacial configuration. This provides an effective strategy to modulate the contact type without altering the electrode material, offering significant flexibility for optimizing device performance in fully 2D architectures.

**Fig. 5:**
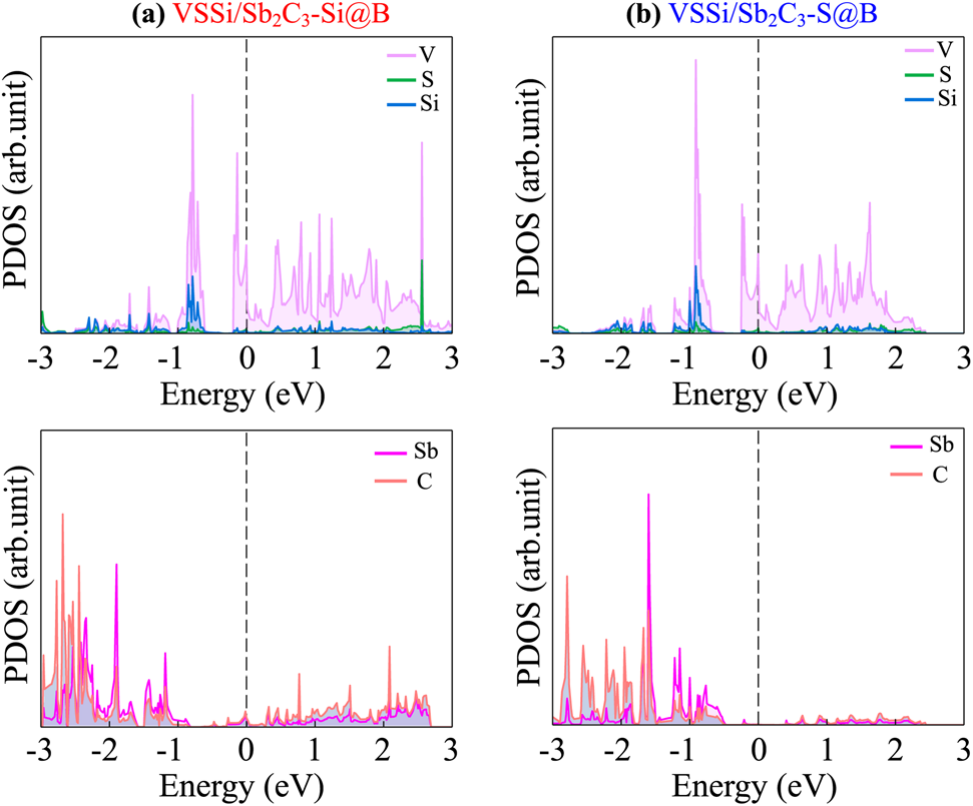
PDOS of all atoms in the VSSi/Sb_2_C_3_ heterostructure with (a) Si-terminated and (b) S-terminated surface. The Fermi level is set to be zero.

To further elucidate the interfacial electronic interactions and validate the contact characteristics, we next analyze the density of states (PDOS) for the most stable configurations of the VSSi/Sb_2_C_3_ with Si-terminated and S-terminated surface, as shown in Fig. 5. For the Si-terminated Si@B stacking, the electronic states originating from VSSi and Sb_2_C_3_ exhibit strong hybridization around the Fermi level, suggesting the emergence of an OhC contact. In contrast, for the S-terminated S@A configuration, the PDOS reveals relatively weak hybridization between the two layers around the Fermi level, and the states of Sb_2_C_3_ remain well separated from *E*_F_, indicating the existence of a Schottky barrier at the interface. Furthermore, negligible metal-induced gap states (MIGS) are observed within the band-gap region, further supporting the Schottky-contact nature of this configuration. This indicates that the penetration of metallic states from the VSSi layer into the band gap of Sb_2_C_3_ is strongly suppressed, a behavior that originates from the weak interfacial coupling. As a result, Fermi level pinning is significantly reduced, and the Schottky barrier height is expected to follow the Schottky–Mott limit more closely.

**Fig. 6:**
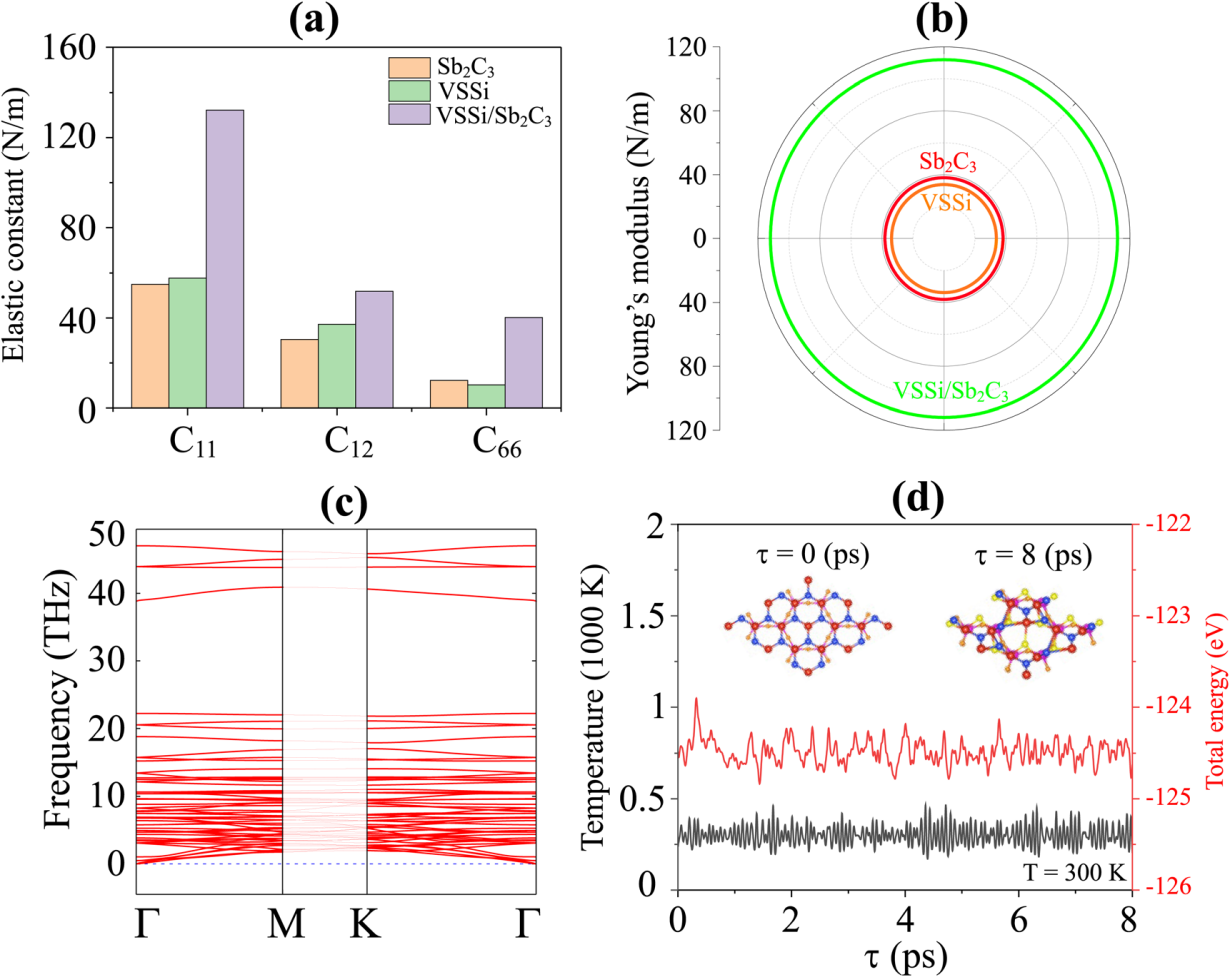
(a) Calculated elastic constants and (b) Young's modulus of the VSSi/Sb_2_C_3_ heterostructure together with those of the individual VSSi and Sb_2_C_3_ monolayers. (c) Phonon band structure and (d) temperature evolution obtained from AIMD simulations of the heterostructure.

Fig. 6 summarizes the elastic response and phonon characteristics of the VSSi/Sb_2_C_3_ heterostructure, which are employed to evaluate its mechanical robustness and dynamical stability. Owing to its hexagonal symmetry, the VSSi/Sb_2_C_3_ heterostructure possesses two independent elastic constants, *C*_11_ and *C*_12_, while the shear modulus *C*_66_ is given by *C*_66_ = (*C*_11_–*C*_12_)/2. The calculated elastic constants for the heterostructure are *C*_11_ = 132.22 N m^−1^, *C*_12_ = 51.87 N m^−1^, and *C*_66_ = 40.18 N m^−1^. Compliance with the Born stability conditions demonstrates the mechanical integrity of the VSSi/Sb_2_C_3_ heterostructure. For comparison, the elastic constants of the individual VSSi and Sb_2_C_3_ monolayers are also included. A comparison of the elastic constants in Fig. 6(a) indicates that the VSSi/Sb_2_C_3_ heterostructure exhibits greater resistance to in-plane deformation than the isolated monolayers, highlighting the beneficial effect of heterostructure formation on mechanical stiffness. Furthermore, the angular dependence of the Young's modulus for the VSSi/Sb_2_C_3_ heterostructure and the constituent monolayers is also evaluated to assess the in-plane mechanical anisotropy. The circular polar plots, as shown in Fig. 6(b), indicate that both the heterostructure and the individual layers exhibit isotropic mechanical behavior within the plane, which is consistent with their hexagonal symmetry. Notably, the VSSi/Sb_2_C_3_ heterostructure exhibits a significantly higher Young's modulus (111.88 N m^−1^) compared to the isolated monolayers (38.08 N m^−1^ for Sb_2_C_3_ and 33.78 N m^−1^ for VSSi), suggesting a substantial enhancement in in-plane stiffness upon heterostructure formation. Moreover, the phonon dispersion curves shown in Fig. 6(c) reveal the absence of imaginary frequencies throughout the Brillouin zone for the VSSi/Sb_2_C_3_ heterostructure, confirming their dynamical stability. To further evaluate the thermal stability of the VSSi/Sb_2_C_3_ heterostructure, AIMD simulations were performed at 300 K, and the corresponding results are presented in Fig. 6(d). It is evident that the total energy fluctuates only slightly around its equilibrium value throughout the 8 ps simulation period, without any abrupt variation or drift. Furthermore, the optimized structure remains intact throughout the simulation, with no evidence of bond dissociation or significant structural distortion. These results indicate that the VSSi/Sb_2_C_3_ heterostructure remains structurally intact under thermal perturbations at room temperature, confirming its excellent thermodynamic stability. The consistency of the mechanical, dynamical, and thermal stability assessments demonstrates the overall robustness of the VSSi/Sb_2_C_3_ heterostructure, suggesting that it can be realistically synthesized and employed in future device technologies.

**Fig. 7:**
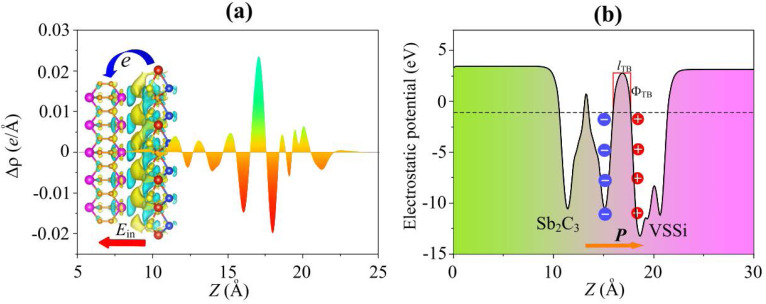
(a) The planar-averaged charge density difference and (b) the electrostatic potential of the VSSi/Sb_2_C_3_ heterostructure.

Additional information regarding interfacial electronic interactions can be obtained from the planar-averaged charge density difference and electrostatic potential distributions shown in Fig. 7. These quantities reveal the charge-transfer characteristics and the development of an internal electric field within the heterostructure. The charge density difference (CDD) is determined according to:

4 Δ*ρ* = ρ_VSSi/Sb_2_C_3__ − *ρ*_VSSi_ − *ρ*_Sb_2_C_3__,

where *ρ*_VSSi/Sb_2_C_3__ denotes the total charge density of the heterostructure, while *ρ*_VSSi_ and *ρ*_Sb_2_C_3__ correspond to the charge densities of the isolated VSSi and Sb_2_C_3_ monolayers, respectively. As presented in Fig. 7(a), the electron depletion (negative Δ*ρ*) occurs primarily in the VSSi layer, while electron accumulation (positive Δ*ρ*) is mainly concentrated in the Sb_2_C_3_ layer. The observed charge redistribution suggests a net electron transfer from the VSSi layer toward the Sb_2_C_3_ layer. Bader charge analysis further confirms this interfacial charge transfer, revealing that approximately 0.025 electrons is transferred from the VSSi layer to the Sb_2_C_3_ layer. As a result, an internal electric field (*E*_in_) is established at the interface, pointing from VSSi toward Sb_2_C_3_. The formation of this built-in *E*_in_ plays a crucial role in governing the carrier transport across the heterointerface. The strength of the built-in electric field can be estimated as? *E*_int_ = Δ*Φ*/*ed*, where Δ*Φ* is the work-function difference between the VSSi and Sb_2_C3 layers, *e* is the elementary charge, and *d* is the equilibrium interlayer separation in the VSSi/Sb_2_C_3_ heterostructure. The built-in electric field is calculated to be *E*_in_ = 0.114 V Å^−1^. Such a relatively strong internal electric field facilitates charge separation and carrier transport across the interface, thereby benefiting the contact performance of the VSSi/Sb_2_C_3_ heterostructure. Specifically, the transferred electrons tend to accumulate near the Sb_2_C_3_ side, forming a potential well that facilitates electron conduction within this region. Meanwhile, the depletion of electrons in the VSSi layer leads to the formation of a space-charge region, which further modulates the band alignment at the interface.

The corresponding electrostatic potential distribution, shown in Fig. 7(b), provides additional insight into the interfacial electric-field characteristics of the heterostructure. A potential step is observed across the interface, indicating the formation of an *E*_in_ induced by interfacial charge redistribution. The lower electrostatic potential of the VSSi layer compared with that of Sb_2_C_3_ promotes electron migration toward the semiconductor layer, giving rise to interfacial charge redistribution and the associated built-in electric field. Furthermore, based on the electrostatic potential profile, the tunneling barrier can be quantitatively characterized by its barrier height (*Φ*_TB_) and barrier width (*l*_TB_). When a metal is interfaced with a semiconductor, an interfacial potential barrier is generally established due to the misalignment between their electronic energy levels. In this case, charge carriers can traverse this barrier *via* quantum mechanical tunneling, enabling carrier transport across the interface even when the barrier height exceeds the thermal energy. Interfacial charge transport is governed not only by the Schottky barrier height but also by the tunneling barrier encountered by carriers. To quantify this effect, the tunneling probability (*P*_TB_) is estimated using:5
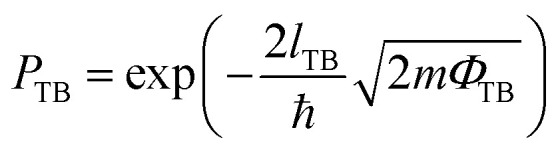
where *m* denotes the free electron mass and ℏ is the reduced Planck constant. Based on the electrostatic–potential profile, the interfacial tunneling barrier is estimated to have a height of 1.77 eV and a width of 3.82 Å for the VSSi/Sb_2_C_3_ heterostructure. Based on these values, the calculated tunneling probability is approximately 3%. This relatively low tunneling probability suggests a moderate quantum tunneling process across the interface. Such a value is comparable to those reported for typical vdW heterostructures involving 2D metal/semiconductor contacts, including TMD-based metal–semiconductor systems,^50^ MoSH/MoSi_2_N_4_ heterostructures,^51^ and graphene-based metal–semiconductor interfaces.^52,53^

Although the tunneling probability offers initial insight into the carrier transmission capability, it does not fully quantify the interfacial transport resistance. To address this, we further evaluate the tunneling-specific resistivity (*ρ*_*t*_) using the Simmons model as follows:6
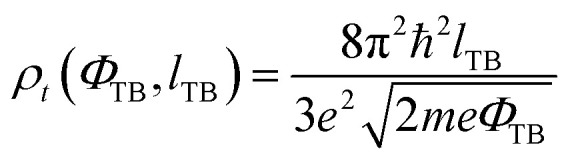


The tunneling-specific resistivity is estimated to be *ρ*_*t*_ = 6.5 × 10^−9^ Ω cm^2^, indicating a relatively low interfacial resistance and efficient carrier transport across the VSSi/Sb_2_C_3_ heterostructure.This value is comparable to those reported for conventional Bi/MoS_2_,^54^ semimetal/TMD,^55^ and graphene-based heterostructures,^52,53^ suggesting that the present system can achieve high-quality electrical contact. Such a low *ρ*_*t*_ further confirms the potential of Janus VSSi as an efficient 2D metallic electrode for forming low-resistance contacts with Sb_2_C_3_, which is highly desirable for next-generation nanoelectronic devices.

**Fig. 8:**
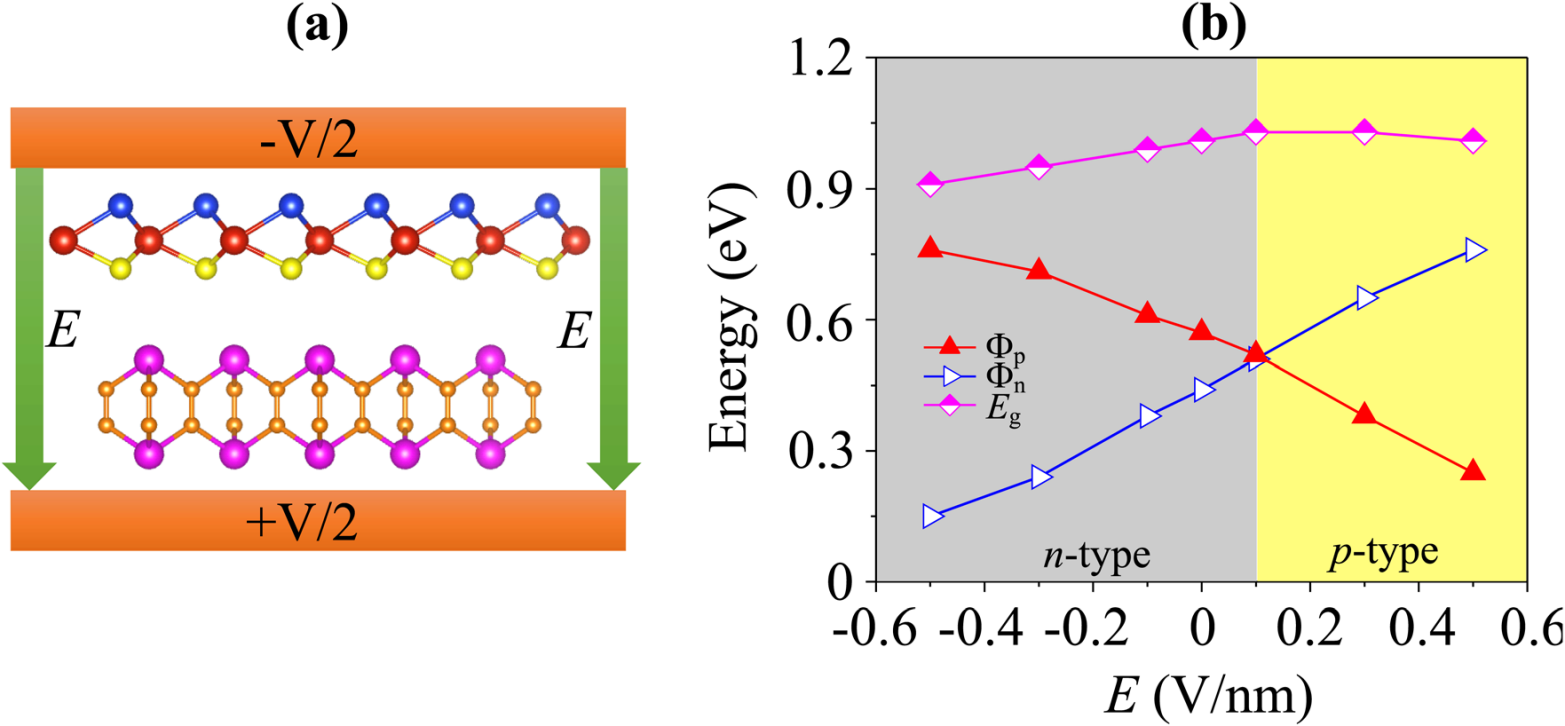
(a) Schematic illustration of applied electric field and (b) the variations in the Schottky barriers of the VSSi/Sb_2_C_3_ heterostructure under applied electric fields.

**Fig. 9:**
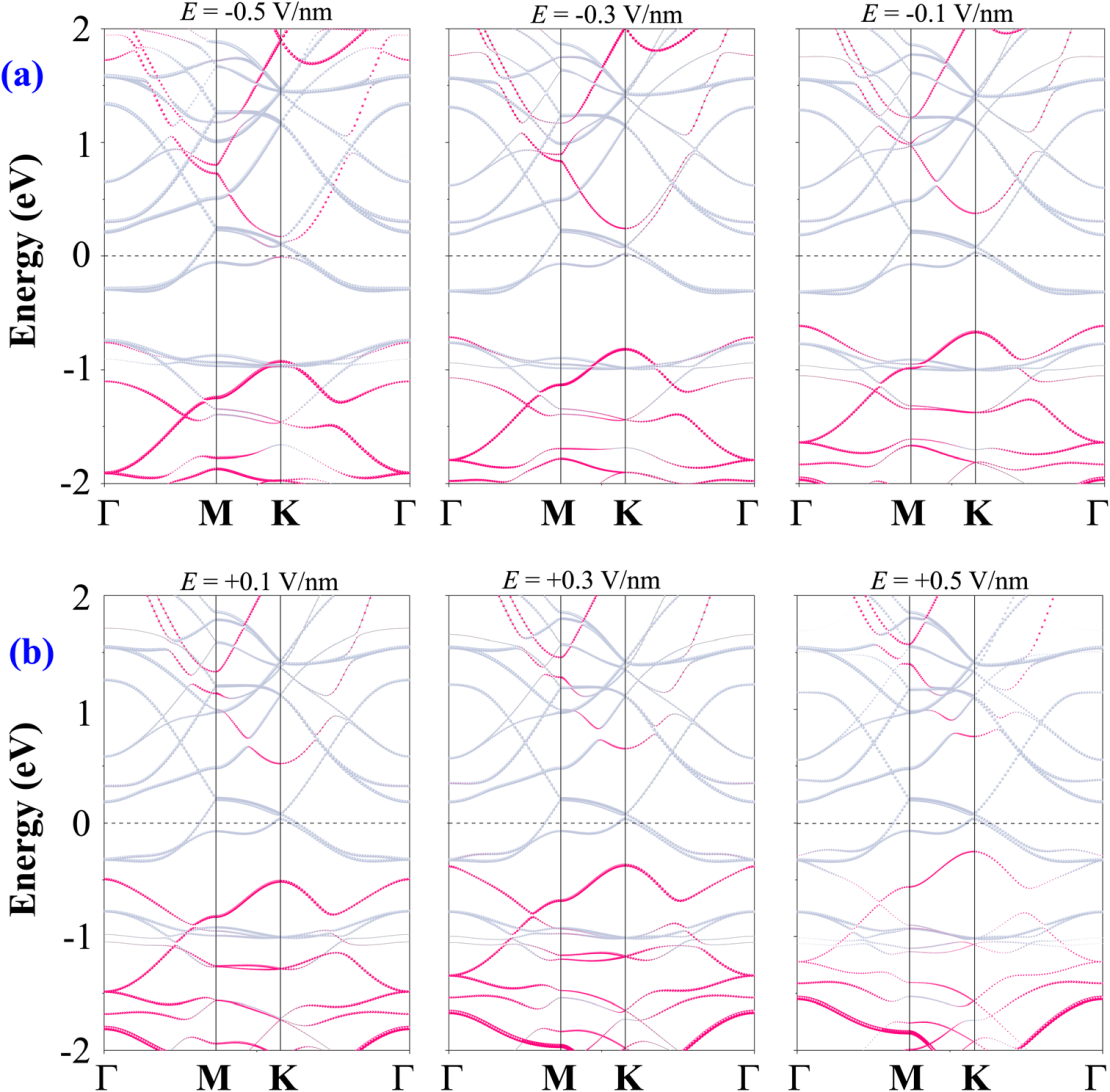
Projected band structures of the VSSi/Sb_2_C_3_ heterostructure under (a) negative and (b) positive electric fields.

Furthermore, electrical-field engineering has emerged as a powerful strategy for controlling the electronic properties of layered vdW systems.^56–59^ To further enhance the understanding and controllability of interfacial contact properties, it is essential to investigate the influence of an external electric field on the Schottky barrier and contact behavior of the VSSi/Sb_2_C_3_ heterostructure. A schematic illustration of the applied electric field is shown in Fig. 8(a). The calculated results reveal a pronounced field-dependent behavior of the Schottky barriers. Applying a negative electric field favors electron injection by lowering *Φ*_n_, while simultaneously suppressing hole injection through an increase in *Φ*_p_. As a result, *Φ*_n_ becomes smaller than *Φ*_p_, indicating that electron injection is more favorable and the heterostructure maintains its n-type Schottky contact. The observed variation in the Schottky barriers originates from the band-structure evolution induced by the external electric field, which alters the energy alignment between the semiconductor band edges and the heterostructure Fermi level, as illustrated in Fig. 9(a). Specifically, the CBM at the *K* point gradually approaches *E*_F_ under a negative electric field, thereby lowering the *Φ*_n_. In contrast, the VBM shifts farther from the Fermi level, giving rise to an increase in the *Φ*_p_.

A markedly different trend is observed when the electric field is applied in the positive direction. In this case, the electron-injection barrier increases, whereas the hole-injection barrier decreases, making hole transport energetically more favorable. As a result, the contact characteristics gradually evolve from an n-type to a p-type Schottky contact. As shown in Fig. 8(b), this conversion occurs at electric-field strengths of *E* ≥ +0.1 V nm^−1^. The underlying mechanism can be understood from the field-induced modification of the band alignment. Increasing the positive electric field drives the valence-band maximum closer to the Fermi level while simultaneously pushing the conduction-band minimum to higher energies. Consequently, the hole barrier is progressively reduced, whereas the electron barrier becomes larger. Once the hole barrier falls below the electron barrier *Φ*_p_ < *Φ*_n_, the dominant carrier injection changes from electrons to holes, marking the transition to a p-type Schottky contact. The continuous evolution of the band edges suggests that the band alignment can be finely tuned by the magnitude of the applied electric field. All these findings demonstrate that electric-field-induced band-edge modulation serves as an effective strategy for engineering interfacial contact properties, enabling a controllable transition between n-type and p-type Schottky contacts.

## 4 Conclusions

In this work, we have systematically investigated the structural, electronic, and interfacial properties of the Janus VSSi/Sb_2_C_3_ vdW heterostructure using first-principles calculations. The results demonstrate that the heterostructure is energetically, mechanically, and dynamically stable, confirming its feasibility for experimental realization. Our results reveals that the contact characteristics strongly depend on the interfacial configuration. The Si-terminated interface forms an ohmic contact, while the S-terminated interface exhibits an n-type Schottky contact with a relatively low Schottky barrier height, favoring efficient electron injection. The absence of significant metal-induced gap states (MIGS) indicates weak Fermi level pinning, allowing the Schottky barrier to closely follow the Schottky–Mott limit. Furthermore, the tunneling probability and tunneling-specific resistivity calculations confirm that the heterostructure possesses low contact resistance and efficient carrier transmission. Importantly, the application of the electric fields enables effective tuning of the Schottky barrier and induces a transition between n-type and p-type contacts. These results establish Janus VSSi as a promising two-dimensional metallic electrode and highlight the VSSi/Sb_2_C_3_ heterostructure as a potential building block for future nanoelectronic and optoelectronic devices.

## Conflicts of interest

There are no conflicts to declare.

## Supplementary Material

RA-016-D6RA03814J-s001

## Data Availability

There is no additional data associated with this article. Supplementary information (SI) is available. See DOI: https://doi.org/10.1039/d6ra03814j.
