# Supplementary material for: Janus VSSi as an efficient 2D electrode for Sb2C3 with tunable Schottky contact: a first-principles study
Source: RSC Adv. 2026 Jul 6;16(35):36932–43. doi: 10.1039/d6ra03814j (PMC13334382; doi:10.1039/d6ra03814j)
Supplement: RA-016-D6RA03814J-s001 [file RA-016-D6RA03814J-s001.pdf]

Supplementary Information (SI) for RSC Advances.
This journal is © The Royal Society of Chemistry 2026

# Supporting Information (SI)
# Janus VSSi as an Efficient 2D Electrode for $Sb_2C_3$ with Tunable Schottky Contact: A First-Principles Study

Ho Kim Dan,[1,2] Huynh Thi Phuong Thuy,[3] Le Phuong Long,[4] Le Dinh Phuoc,[5] Nguyen D. Hien[6] and Khang D. Pham[7,8,†]

[1]*Optical Materials Research Group, Science and Technology Advanced Institute, Van Lang University, Ho Chi Minh City, Vietnam.*

[2]*Faculty of Applied Technology, School of Technology, Van Lang University, Ho Chi Minh City, Vietnam*

[3]*Thu Dau Mot University, Ho Chi Minh City, Viet Nam*

[4]*Center of Scientific Research and Application, Lac Hong University, No. 10 Huynh Van Nghe Str, Tran Bien Ward, Dong Nai Province, Vietnam.*

[5]*Faculty of Electricity, Electronics and Material Technology, University of Sciences, Hue University, Hue, Vietnam*

[6]*Nha Trang Center Ethnic Minority Pre-University School, No. 46 Nguyen Thien Thuat Str., Nha Trang Ward, Khanh Hoa Province, Vietnam*

[7]*Institute of Research and Development, Duy Tan University, Da Nang 550000, Vietnam*

[8]*School of Engineering & Technology, Duy Tan University, Da Nang 550000, Vietnam*

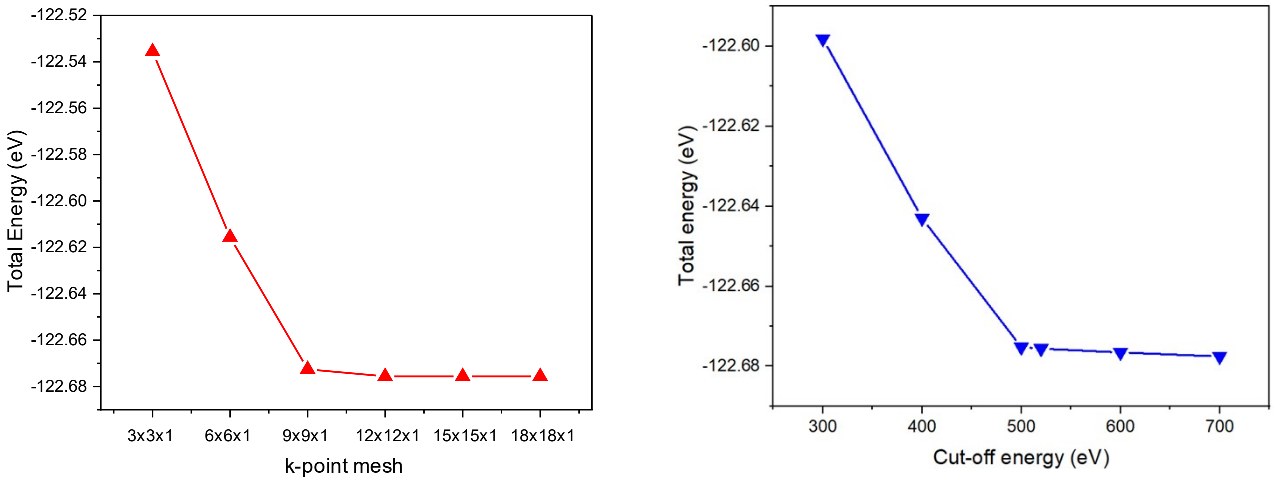


Fig. S1. The convergence tests for the *k*-point mesh and cut-off energy of the VSSi/$Sb_2C_3$ heterostructure.
